# Youth Group Engagement in Noncompliant Communities During Supplemental Immunization Activities in Kaduna, Nigeria, in 2014

**DOI:** 10.1093/infdis/jiv510

**Published:** 2016-04-02

**Authors:** Audu Musa, Pascal Mkanda, Fadinding Manneh, Charles Korir, Charity Warigon, Emmanuel Gali, Richard Banda, Gregory Umeh, Peter Nsubuga, Ana Chevez, Rui G. Vaz

**Affiliations:** 1 World Health Organization, Country Representative Office, Abuja, Nigeria; 2 World Health Organization, Regional Office for Africa, Brazzaville, Congo; 3 Global Public Health Solutions, Atlanta, Georgia

**Keywords:** youth engagement, polio supplemental immunization activities, OPV-refusing households

## Abstract

***Introduction.*** One of the major challenges being faced in the Global Polio Eradication Initiative program is persistent refusal of oral polio vaccine (OPV) and harassment of vaccination team members by youths. The objective of the study was to describe the strategy of collaborating with recognized youth groups to reduce team harassment during vaccination campaigns and improve vaccination coverage in noncompliant communities.

***Methods.*** We assessed data from polio vaccination activities in OPV-refusing communities in the Igabi and Zaria local government areas (LGAs) of Kaduna State in Nigeria. We evaluated the following factors to determine trends: enhanced independent monitoring data on the proportion of children missed by vaccination activities (hereafter, “missed children”), lot quality assurance surveys, and vaccination team harassment.

***Results.*** The proportion of missed children decreased in both LGAs after the intervention. In Igabi LGA and Zaria LGA, the lowest proportions of missed children before and after the intervention decreased from 7% to 2% and from 5% to 1%, respectively. Lot quality assurance survey trends showed an improvement in immunization coverage 1 year after youth groups' engagement in both LGAs.

***Conclusions.*** Systematic engagement of youth groups has a great future in polio interruption as we approach the endgame strategy for polio eradication. It promises to be a veritable innovation in reaching chronically missed children in OPV-refusing communities.

Tremendous progress to a poliomyelitis free world has been made since the World Health Assembly in 1988 inaugurated the Global Polio Eradication Initiative [1], with >99% reduction in confirmed cases of wild poliovirus (WPV) [2]. Since this drive to eradicate poliomyelitis, 3 countries—Nigeria, Afghanistan, and Pakistan—remain endemic reservoirs of WPV [3]. Poliomyelitis is one of the leading causes of acute flaccid paralysis among individuals aged <5 years in developing countries [4, 5]. Supplementary immunization activities (SIAs) are aimed at delivering potent oral polio vaccine (OPV) to children aged <5 years, using various teams to vaccinate children at homes, on the street, at transit points, and at health facilities. However, in some cases this strategy is fraught with challenges, including harassment of vaccination team members and refusal of OPV, resulting in children who are chronically missed by SIAs [6].

Youth groups worldwide are often involved in promoting peace and harmony, but sometimes they are used to instigate unrest and violence in the society [7, 8]. Systematic youth group engagement is one of the pillars of the Indian success story in polio eradication and was particularly useful in high-risk settlements, notorious for health worker harassment and abuse [9, 10, 11]. Pakistan, like Nigeria, has experienced hostility toward and attacks of vaccination teams, necessitating diverse innovations, such youth group involvement, to reach chronically missed children, especially in the Federally Administered Tribal Areas and Khyber Pakhtunkhwa province [12, 13, 14].

Kaduna State is located in the northwestern region of Nigeria. The state has made tremendous progress in interrupting poliovirus transmission over the years. The risk of polio transmission is very high in Kaduna State, based on the Nigeria National Emergency Operational Center risk categorization of 2014, and has 13 local government areas (LGAs) at very high risk. In November 2012, Kaduna State recorded 15 WPVs (second highest to Kano, which had the highest number of cases in the country), all within the 13 LGAs at very high risk. Although the last WPV case in the state was in November 2012, the state recorded recovery of another WPV type 1 during environmental surveillance in April 2014, in Zaria LGA, and reported recovery of 1 circulating vaccine derived poliovirus (cVDPV) during environmental surveillance in week 23 from Rigasa in Igabi LGA and intermittent recovery of cVDPV from 2 environmental surveillance sites in Zaria LGA from week 24 to week 47 of 2014, which was an indication of low population immunity [15, 16].

In 2014, security threats to vaccination team members were prominent in some part of Kaduna State, especially Igabi, Kaduna North, Zaria, Giwa, Soba, and Kudan LGAs [17, 18]. On several occasions, apart from OPV vaccination refusal, team members were assaulted and immunization materials destroyed, with a resultant increase in the number of children chronically missed by SIAs (hereafter, “missed children”) [19].

To improve the effectiveness of SIA implementation and sustain Nigeria's goal of interrupting WPV transmission, the 27th Expert Review Committee directed states to develop special plans to improve the quality of performance in high-risk LGAs and areas of high insecurity and to reduce the number of chronically missed children during SIAs [20]. Youth engagement has significantly reduced harassment of vaccination team workers, especially teams that directly observe oral polio vaccination (DOPV), and has allowed health workers to operate freely both in OPV-refusing settlements and security-compromised settlements. The engaged youths were identified from several areas, including existing youth associations in the affected communities, motorcycle riders (known as *Achabas*), local vigilante groups, and Motor park tout (someone who provides services at the motor park but not formally engaged). The youths are engaged on the basis of a situational analysis and mapping of OPV-refusing communities, and after orientation they are assigned to accompany vaccination members working in high-risk and noncompliant settlements where intimidation, harassment, and abuse of the polio SIA vaccination team is possible. The systematic engagement of youth groups was piloted in the Rigasa settlement of Igabi LGA, Kaduna State, which was known for polio vaccination refusal and security threats to vaccination team members. The innovation was introduced in May 2014 but perfected and scaled up in July 2014 to include 8 other LGAs in Kaduna and Sokoto states. We describe the strategy of collaborating with recognized youth groups to reduce SIA team harassment during vaccination campaigns. We also assess the trend in the numbers of missed children and noncompliant households in communities where the potential for noncompliance is known to be increased, before and after the innovation of the systematic engagement of youth groups. In addition, we assess changes in trends of poliovirus detection in the vulnerable communities in which the intervention was implemented.

## METHODS

### Description of Organizational Structure of Youth Engagement in Polio Eradication Initiative (PEI) Activities

Youths in these 2 LGAs had a youth forum, which was coordinated at the LGA and settlement levels through the appointed leaders at the respective levels. The leadership of the youth groups worked in collaboration with security agents and traditional leaders.

### Process of Operations

The intervention took place before, during, and after the campaign.

Prior to youth involvement in polio vaccination campaign, we developed operational protocols with the Nigeria Field Support Unit, Nigeria Country Office, World Health Organization (WHO). Planning meetings were held to list vulnerable settlements, using noncompliance and monitoring data, as well as reports from senior supervisors that were obtained from daily review meetings during previous rounds of vaccinations. We selected the youths in collaboration with the LGA youth department, leveraging the existing youth-associated community-based organizations. These organizations, with the support of traditional leaders, assisted us in selecting other influential youths, as well as youths who were thought to have been directly linked with OPV resistance and team harassment. In preparation for their sensitization, relevant information, education, and communication and sensitization materials were developed. Sensitization sessions were conducted for 1 day with all of the listed youths, using lecture, discussion, and role-play. The training agenda included an overview of PEI activities in Nigeria, the journey so far, and challenges. We also included highlights of the poliomyelitis burden and reason for targeting polio for eradication. The lists of noncompliant households and communities with documented hostility to OPV vaccination teams were shared with respective youth groups, based on their area of familiarities and local influence.

During the polio vaccination campaign, the sensitized youth were deployed to areas of documented noncompliance and vaccination team harassment. The youths worked with the vaccination teams for 7–8 days (2–3 days for DOPV, 4 days for house-to-house vaccination, and 1 day for mop-up activities) to accompany teams in high-risk or hostile communities. They supported the vaccination team members in carrying and distributing pluses, as well as crowd control during DOPV and at health camps. They also, in some instances, provided entertainment to the community during DOPV. The LGA youth leader and cluster or settlement youth leaders supervised the activities of members through regular telephone calls and routine checks, and they were complemented by senior supervisors from the state and the partners assigned to the LGA or ward. For any incidents or reports of threat or harassment of any team members, the cluster or settlement youth leader immediately contacted the LGA PEI youth leader, and they jointly attempted to resolve the issue. However, in cases of violence or security threats beyond the capacity of the youth leaders to handle, traditional and security agents are invited for intervention.

For effective intra-campaign review, daily evening review meetings with polio SIA personnel were conducted at ward and LGA levels, during which WHO staff in the respective LGAs collated issues, challenges, and data.

After each polio vaccination campaign, data from the affected communities were collated and analyzed. Feedback and debriefing were organized with the youth leaders in attendance, together with representatives of traditional leaders.

We evaluated the impact of the intervention by using qualitative and quantitative methods. Data used were from supervisory reports, noncompliant households, tally sheets, and monitoring activities, obtained from WHO Kaduna field office. Data were obtained from end-process outside household monitoring, lot quality assurance surveys, and record of noncompliance households trends of team harassments obtained from intra-campaign LGA level daily review meeting. We used data from 8 rounds of SIAs 1 year before the intervention (April 2013, May 2013, June 2013, July 2013, September 2013, December 2013, January 2014, and March 2014) and 1 year after the intervention (July 2013, August 2014, September 2014, October 2014, November 2014, December 2014, January 2015, and March 2015).

### Statistical Analysis

Data were analyzed using SPSS, version 20. The independent *t* test was used to assess the impact of youth groups' involvement in house-to-house polio vaccination to reach chronically missed children in high-risk areas during SIAs in Kaduna State, before and after the intervention.

## RESULTS

The proportion of missed children, as shown in Table 1, decreased in Igabi LGA in all 8 rounds of the SIAs except round 8. The lowest proportion of missed children before the intervention was 7%, while the lowest proportion of missed children after the intervention was 2%. For Zaria LGA, the proportion of missed children decreased in all the rounds after the intervention, compared with before the intervention. The lowest proportion of missed children before the intervention was 5%, compared 1% after the intervention.

**Table 1.** Percentage of Children Missed by Polio Vaccination Activities (End Process) Before and After Youth Group Engagement in Local Government Areas (LGAs) of Zaria and Igabi—Kaduna, Nigeria, 2013–2015

LGA	Igabi, Children Missed, %		Zaria, Children Missed, %	
	Before	After	Before	After
Round 1	15	13	13	5
Round 2	17	13	7	3
Round 3	23	12	12	1
Round 4	8	2	7	1
Round 5	17	11	5	5
Round 6	7	11	6	2
Round 7	13	12	8	4
Round 8	14	19	14	12

Lot quality assurance survey data showed a reduction of nonaccepted vaccination results after the intervention (Table 2). During the 8 rounds assessed as shown in Table 3, in Igabi LGA, the 4126 noncompliant households in round 3 decreased to 778 after the intervention. The highest number of noncompliant households before the intervention was 4126, while the highest number after the intervention was 1766. For Zaria LGA, the number of noncompliant households decreased to 81 from 489 before the intervention.

**Table 2.** Lot Quality Assurance Survey Trends Before and After Youth Group Engagement in Local Government Areas (LGAs) of Zaria and Igabi—Kaduna, Nigeria, 2013–2015

LGA	Igabi		Zaria	
	Before	After	Before	After
Round 1	17	3	23	3
Round 2	16	0	31	2
Round 3	20	0	19	0
Round 4	24	5	4	3
Round 5	5	0	10	3
Round 6	12	0	18	2
Round 7	4	0	5	2
Round 8	9	2	9	6

0–3 accepted with coverage >90%. 4–8 rejected with coverage >80% <90%. 9–19 rejected with coverage >60% <80%. 20 & above rejected with coverage <60%.

**Table 3.** Noncompliance With Polio Immunization Activities Among Households Before and After Youth Group Engagement—Kaduna, Nigeria, 2013–2015

LGA	Igabi		Zaria	
	Before	After	Before	After
Round 1	796	1108	489	512
Round 2	1824	1282	431	119
Round 3	4126	778	311	129
Round 4	2554	1434	242	125
Round 5	2601	1434	220	81
Round 6	919	1766	227	89
Round 7	1993	1559	160	120
Round 8	810	1709	200	148

Abbreviation: LGA, local government area.

Statistical analysis of monitoring data (percentage of missed children), as shown in Table 1, using the paired *t* test, the mean percentage (±SD) was 11.6% ± 5.0% before the intervention and 7.9% ± 5.6% after the intervention. The *P* value was .002, indicating a significant decline in the percentage of missed children.

As shown in Table 4, team harassment per round decreased from >10 incidents to <5 incidences in the ward. In November and December, there was no record of incidents of team harassment.

**Table 4.** Trend of Team Harassment, by Immunization Plus Day (IPD) Round, in Rigasa Ward of Igabi Local Government Area—Nigeria, January–December 14

IPD Round	Harassment Episodes, No.
November 13	13
December 13	10
January 14	12
March 14	9
April 14	11
May 14	8
June 14	4
July 14	3
August 14	2
September 14	2
October 14	2
November 14	0
December 14	0

At the 3 sites where environmental samples were collected weekly, cVDPV was isolated at the Rigasa River sample site in week 23 of 2014 but was not recovered after systematic engagement of youth groups, whereas at the 2 sites in Zaria and Rigasa, Igari, LGAs continued to isolate environmental cVDPVs.

## DISCUSSION

We found that youth groups' engagement significantly enhanced the ability of vaccination teams to vaccinate chronically missed children in OPV-refusing communities of Rigasa and Tudun Wada communities in the Igabi and Zaria LGAs, respectively, of Kaduna State. The presence of the youths gave assurance and a sense of security to vaccination teams, making it easier to reach chronically missed children inside households during house-to-house vaccination and outside households during DOPV. The innovation greatly improved operations and the quality of SIAs in very high-risk communities in Kaduna State. Youth engagement is an innovation that, over time, has gained acceptance across stakeholders and enhanced vaccination team safety in OPV-refusing communities in Kaduna State.

We also found that youth engagement reduced the threat and harassment to vaccination team members. The teams were more confident to inquire about the 6 interpersonal communication skill messages (number of children in the household aged <5 years; whether any children were absent, sick, or sleeping; and whether any visiting child or resident child had sudden weakness of any of the limbs), more assertive in convincing mothers and caregivers to accept OPV, and less fearful in marking households. The incidents of vaccination team harassment have greatly decreased with youth groups' engagement in these 2 LGAs. The youth groups, after undergoing orientation, act as so-called polio ambassadors and, over and above protecting vaccination teams, sensitize fellow youths and sometimes assist teams in persuading their colleagues to allow children in their wards to be vaccinated with OPV [20].

In addition, we also found a positive outcome in terms of reduced poliovirus detection in the affected settlements. There has been no reported cVDPV recovery from environmental samples in Rigasa, Igabi LGA, since the implementation of youth group engagement, although more studies will be needed to support it.

The systematic engagement of youth groups in SIAs in the 2 LGAs involved detailed planning, advocacy visits, supervision, and monitoring. Youth groups in the 2 OPV-refusing communities were identified and contacted. A workable partnership was reached between the leadership of the youth groups and the LGA authorities, and over time modalities of their engagement in vaccination-related activities were agreed upon. The youth groups' hierarchical structure from the cluster level to the ward and LGA levels made coordination and engagement of their members to accompany vaccination teams easy. Youth groups' engagement has been practiced in other countries, such as India and Pakistan, and studies showed that it greatly improved the quality of SIAs and reduced vaccination team harassment and violence. A study of factors responsible for missed vaccination during campaigns identified the unwillingness or inability of health workers to vaccinate because of security concerns as another important rea­son that children remain unvaccinated [21].

The main limitation in interpreting our data is that, because youths were not directly vaccinating children and there were other interventions, such as DOPV, in the targeted communities, the results do not represent the effect of youth engagement alone. In addition, the data could not sufficiently quantify the level of reduction in harassment and abuse, as there was no consistent documentation of this information, except from the extract of the report from the intra-campaign daily review meetings. However, we believe that systematic youth engagement in areas where there is vaccination team harassment may be one innovative intervention that can enable the performance of other vaccination activities.

We recommend that the innovation should be replicated in other OPV-refusing communities in high-risk LGAs and states with documentation of team harassments. Other polio-endemic countries with documented harassment of teams could also adopt systematic youth engagement. The operationalization of this strategy must be constantly reviewed and documented in standard operating procedures, guided by the polio SIA.

In conclusion, systematic engagement of youth groups has great future in polio interruption as we approach the endgame. It promises to be a veritable innovation in reaching chronically missed children in OPV-refusing communities in Nigeria and other polio-endemic countries.

## References

[1] World Health Organization, United Nations Children's Fund. Global immunization data. WHO vaccine-preventable diseases: monitoring. 2011 http://www.who.int/gho/immunization/en/index.html. Accessed 27 July 2015.

[2] Grassly NC The final stages of the global eradication of poliomyelitis. Philos Trans R Soc Lond B Biol Sci 2013; 368:20120140. 23798688 10.1098/rstb.2012.0140 PMC3720038

[3] Abimbola S, Malik AU, Mansoor GF The final push for polio eradication: addressing the challenge of violence in Afghanistan, Pakistan, and Nigeria. PLoS Med 2013; 10:e1001529. 24115915 10.1371/journal.pmed.1001529 PMC3792994

[4] Centers for Disease Control and Prevention. Progress toward eradication of polio—worldwide, January 2011–March 2013. MMWR Morb Mortal Wkly Rep 2013; 62:335–8. 23636027 PMC4604928

[5] Mihigo R, Anya B, Okeibunor J et al African vaccination week as a vehicle for integrated health service delivery. BMC Health Services Res 2015; 15:358. 10.1186/s12913-015-0989-7 PMC4557633 26328630

[6] Saint-Victor DS, Omer SB Vaccine refusal and the endgame: walking the last mile first. Philos Trans R Soc Lond B Biol Sci 2013; 368:20120148. 23798696 10.1098/rstb.2012.0148 PMC3720046

[7] Stoneman D The role of youth programming in the development of civic engagement. Appl Dev Sci 2002; 6:221–6.

[8] Warrs RJ, Flanagan C Pushing the envelope on youth civic engagement: A developmental and liberation psychology perspective. J Community Psychol 2007; 35:779–92.

[9] Hussain RS, McGarvey ST, Shahab T, Fruzzetti LM Fatigue and fear with shifting polio eradication strategies in India: a study of social resistance to vaccination. PLoS One 2012; 7:1–8. 10.1371/journal.pone.0046274 PMC3458871 23050003

[10] John TJ, Vashishtha VM Eradicating poliomyelitis: India's journey from hyperendemic to polio-free status. Indian J Med Res 2013; 137:881–94. 23760372 PMC3734678

[11] Paul Y. Why polio has not been eradicated in India despite many remedial interventions? Vaccine 2009; 27:3700–3. 19464552 10.1016/j.vaccine.2009.03.078

[12] Shah M, Khan M, Shakeel S et al Resistance of polio to its eradication in Pakistan. Virol J 2011; 8:457. 21962145 10.1186/1743-422X-8-457 PMC3192783

[13] Olufowote JO Local resistance to the global eradication of polio: newspaper coverage of the 2003–2004 vaccination stoppage in Northern Nigeria. Health Commun 2011; 26:743–53. 21667367 10.1080/10410236.2011.566830

[14] Alexander JP, Zubair M, Khan M, Abid N, Durry E Progress and peril: poliomyelitis eradication efforts in Pakistan, 1994–2013. J Infect Dis 2014; 210(suppl 1):S152–61. 25316830 10.1093/infdis/jiu450 PMC6261452

[15] Jenkins HE, Aylward RB, Gasasira A et al Implications of a circulating vaccine-derived poliovirus in Nigeria. N Engl J Med 2010; 362:2360–9. 20573924 10.1056/NEJMoa0910074

[16] Okonko IO, Ogun AA, Adedeji AO, Akanbi OA, Udeze AO, Motayo OB Circulating vaccine-derived poliovirus and its implications for polio surveillance and eradication in Nigeria: A review of the literature. Sci Res Essays 2009; 4:398–418.

[17] Chang HJ The politics of polio in Northern Nigeria. JAMA 2011; 306:556–7.

[18] Obadare E A crisis of trust: history, politics, religion and the polio controversy in Northern Nigeria. Patterns Prejudice 2005; 39:265–84.

[19] Chen C Rebellion against the polio vaccine in Nigeria: implications for humanitarian policy. Afr Health Sci 2004; 4:205–7. 15687078 PMC2688336

[20] Camino L, Zeldin S From periphery to center: pathways for youth civic engagement in the day-to-day life of communities. Appl Dev Sci 2002; 6:213–20.

[21] Weiss WM, Winch PJ, Burnham G Factors associated with missed vaccination during mass immunization campaigns. J Heal Popul Nutr 2009; 27:358–67. 10.3329/jhpn.v27i3.3378 PMC2761796 19507751

